# The Protection Level of S-RBD SARS-CoV-2 Immunoglobulin G Antibodies Using the Chemiluminescent Immunoassay Compared to the Surrogate Virus Neutralization Test Method

**DOI:** 10.3390/diagnostics14161776

**Published:** 2024-08-14

**Authors:** Agnes Rengga Indrati, Erinca Horian, Nina Susana Dewi, Nida Suraya, Marita Restie Tiara, Hofiya Djauhari, Bachti Alisjahbana

**Affiliations:** 1 Departement of Clinical Pathology, Hasan Sadikin Hospital, Faculty of Medicine, Universitas Padjadjaran, Bandung 40161, West Java, Indonesia; erincahorian@gmail.com (E.H.); rninasdewi@gmail.com (N.S.D.); nidasuraya@yahoo.com (N.S.); 2 Research Center for Care and Control of Infectious Disease, Universitas Padjadjaran, Bandung 40161, West Java, Indonesia; marita.restie@gmail.com (M.R.T.); hofiya@gmail.com (H.D.); b.alisjahbana@gmail.com (B.A.); 3 Departement of Internal Medicine, Hasan Sadikin Hospital, Faculty of Medicine, Universitas Padjadjaran, Bandung 40161, West Java, Indonesia

**Keywords:** antibody protective, IgG SARS-CoV-2 S-RBD, CLIA, SVNT

## Abstract

COVID-19 infection in high-risk populations is fatal and has a poor prognosis, necessitating a test to determine the protectiveness of immune response. Antibody testing is necessary to determine the body’s immune response to COVID-19 infection and also vaccination strategies. Among the various methods available, the chemiluminescent immunoassay (CLIA) test is more widely used and accessible to determine antibody levels. This study aimed to determine the protection level of S-RBD SARS-CoV-2 IgG using CLIA compared to the Surrogate Virus Neutralization Test (SVNT). The population of this study comprised all healthcare professionals who experienced S-RBD SARS-CoV-2 IgG antibody level examinations. S-RBD SARS-CoV-2 IgG antibody levels were examined using CLIA and SVNT. The cut-off was determined using a receiver operating characteristic (ROC) curve, and area under the curve (AUC) measurements were evaluated. The result showed a strong positive correlation between S-RBD SARS-CoV-2 IgG CLIA and SVNT, with a value of r = 0.933 and *p* < 0.001. The value ≥ 37.29 BAU/mL was determined as the cut-off based on SVNT 30% inhibition level with sensitivity, specificity, and positive and negative predictive values of 96.5%, 90.9%, 96.5%, and 90.9%, respectively. A titer of antibodies greater than or equal to 37.29 BAU/mL with CLIA showed the presence of protective antibodies compared to SVNT.

## 1. Introduction

COVID-19 infection in high-risk populations is fatal and has a poor prognosis, necessitating a test to determine the protectiveness of immune response. Even though the COVID-19 pandemic has passed, high-risk populations still need to be protected from COVID infection, for example, through vaccination. Antibody testing is necessary to determine the body’s immune response to COVID-19 infection and also vaccination strategies [1]. Immune responses to vaccination are routinely measured in blood for cellular immune responses and serum for humoral immune responses. Cell-mediated immune responses are measured by quantifying the number of sub-sets of lymphocyte populations, for example, flow cytometry analysis of CD4 and CD8 levels, and functional assays, for example, the interferon gamma release assay. Humoral immune responses are measured by immunoassays (e.g., quantifying IgM and IgG antibody levels or titers using ELISA) and functional assays (e.g., neutralizing antibody bioassays) [2].

In the context of SARS-CoV-2, IgA, IgM, and IgG antibody, ELISA assays using plasma or serum are employed to identify individuals with an adaptive immune response to SARS-CoV-2, indicating recent or past infection. During the early stages of infection, typically 5–7 days after symptom onset, IgM antibodies are usually detected. IgG antibodies appear during the active and late phases of infection or during recurrent infections. A small percentage of antibodies bind to sites on the virus that interact with host proteins, masking these sites and preventing the virus from entering host cells. These antibodies are known as neutralizing antibodies. The primary target for neutralizing antibodies on coronaviruses is the spike (S) protein, a homo-trimeric glycoprotein embedded in the viral membrane. Potent neutralizing antibodies often target the receptor binding site in the S1 subunit, blocking interactions with the host receptor and preventing viral entry into the cell [2]. Kenny et al. demonstrate the feasibility of using a binding IgG threshold as a surrogate for neutralizing capacity after vaccination, offering the potential for the use of a simplified laboratory assay to determine host immunity to SARS-CoV-2. Neutralizing antibodies against SARS-CoV-2 correlate with anti-spike IgG binding antibodies [3].

Neutralizing antibodies are key biomarkers of humoral immunity and vaccine effectiveness. Inducing a neutralizing antibody response is a primary objective for many vaccine development programs, as it correlates with disease protection. To understand immunity after natural infection or vaccination, a functional analysis of the elicited antibody responses, such as avidity for the most immunogenic viral antigens and virus neutralizing activity, is of utmost importance [4]. For SARS-associated coronaviruses, three types of virus neutralization assays are commonly referenced in the literature. These assays use a dilution series of serum samples from infected patients or animals to measure the level (or titer) of neutralizing antibodies present. The cytopathogenic effect-based (CPE) virus neutralization assay assesses neutralization by visually grading virus-infected or uninfected cells. The plaque reduction neutralization assay (PRNT), considered the gold standard for evaluating neutralizing antibodies, quantifies virus neutralization by counting plaques [5]. The gold standard for detecting and measuring neutralizing antibody is the Virus Neutralization Test (VNT). However, the VNT has limitations, including the requirement for handling live SARS-CoV-2, cell culture in the process, high biosafety laboratory (BSL) level 3, a considerable amount of time, and skilled operators. Antibody protection levels refer to the levels considered sufficient to provide protection against disease, in this case, COVID-19 [6].

An alternative method to measure neutralizing antibodies is the Pseudovirus-based Virus Neutralization Test (PVNT) conducted at BSL level 2, which uses non-infectious virus, such as Lentivirus. However, PVNT requires a significant amount of time and skilled operators [6]. To overcome the limitations of VNT and PVNT, the Surrogate Virus Neutralization Test (SVNT) was developed [7]. This test can detect neutralizing antibodies without using a live virus or cell and can be completed in 1–2 h in a BSL level 2 laboratory [7,8]. The test mimics the interaction between the virus and host cell by simulating S-RBD antibody in a reaction tube and ACE2 receptor on the solid phase of ELISA. According to previous studies, the specific interaction between S-RBD antibody and ACE2 receptor could then be neutralized and blocked by neutralizing antibodies in the subject’s serum, similar to the conventional VNT [9,10].

The current challenge is understanding the mechanism through which COVID-19 infection and vaccination provide effective immunity, influence the severity of clinical manifestations, and inform strategies. A previous study showed that the SVNT test was a valuable tool for assessing the protective immunity to SARS-CoV-2, specifically in the context of vaccination campaigns and monitoring the spread of the virus in the population [7]. The CLIA test is widely used to determine antibody levels due to the accessibility, specifically in Indonesia as a developing country. However, this test has not indicated protective immunity despite its widespread use. Therefore, this study aimed to evaluate the protective antibody levels against SARS-CoV-2 S-RBD using the CLIA compared to the SVNT method.

## 2. Materials and Methods

### 2.1. Study Design

This cross-sectional study was conducted using clinical and demographic data of subjects, including gender, age, history of COVID-19 infection and vaccination, comorbidities, as well as height and weight, obtained from medical records. The population comprised healthcare workers who had an S-RBD SARS-CoV-2 IgG antibody levels examination from May to August 2021 at Hasan Sadikin General Hospital, also serving as the inclusion criteria. The exclusion criteria were healthcare workers who had an S-RBD SARS-CoV-2 IgG antibody levels examination with sample conditions of hemolysis, icteric, or lipemic. Blood plasma samples were examined to determine S-RBD SARS-CoV-2 IgG levels using Siemens ADVIA Centaur^®^ CLIA (Munich, Germany) and GenScript cPASS^TM^ SVNT (Piscataway, NJ, USA).

### 2.2. Ethical Clearance

This study was approved by the Health Research Ethics Committee of Hasan Sadikin General Hospital, Universitas Padjadjaran with the number 410/UN6.KEP/EC/2021 on 17 May 2021. This study was conducted in accordance with the Declaration of Helsinki and all data were kept anonymous.

### 2.3. Chemiluminescent Immunoassay Anti-SARS-CoV-2 IgG Antibody Test

The CLIA anti-SARS-CoV-2 IgG antibody test was performed according to the manufacturer’s instructions [11]. The ADVIA Centaur sCOVG assay is a fully automated 2-step sandwich immunoassay using indirect chemiluminescent technology. Serum and plasma (lithium heparin) are the recommended sample types for this assay. This assay requires 40 μL of sample for a single determination. This volume does not include the unusable volume in the sample container or the additional volume required when performing duplicates or other tests on the same sample. The solid phase contains a preformed complex of streptavidin-coated microparticles and biotinylated SARS-CoV-2 recombinant antigens. The antigen-coated particles subsequently capture SARS-CoV-2 specific antibodies in the specimen. Furthermore, the antibody-antigen complex is washed, followed by the addition of Lite Reagent, which consists of an acridinium-ester-labeled anti-human IgG mouse monoclonal antibody. The entire complex is washed to generate a signal in the presence of a Lite Reagent bound to the solid phase through the anti-SARS-CoV-2 IgG:SARS-CoV-2 antigen complex.

### 2.4. SARS-CoV-2 Neutralization Antibody Detection Kit

The SARS-CoV-2 Neutralization Antibody Detection Kit (Genscript Biotech, Leiden, The Netherlands) was performed according to the manufacturer’s instructions [12]. The kit is a blocking ELISA detection tool, which mimics the virus neutralization process. The kit contains two key components: the horseradish peroxidase (HRP) conjugated recombinant SARS-CoV-2 RBD fragment (HRP-RBD) and the human ACE2 receptor protein (hACE2). The protein–protein interaction between HRP-RBD and hACE2 can be blocked by neutralizing antibodies against SARS-CoV-2 RBD. Serum samples, as well as negative and positive controls, were diluted at a ratio of 1:10 in buffer, mixed at 1:1 with an HRP-RBD working solution, and incubated at 37 °C for 30 min. Subsequently, 100 μL of samples and controls was added into the wells of a 96-well plate coated with the ACE2 receptor protein. The plate was incubated at 37 °C for 15 min and washed 4 times with 300 μL washing buffer. This was followed by the addition of 100 μL substrate solution, and the plate was incubated in the dark for 15 min at RT. Finally, 50 μL stop solution was added per well, and the absorption at 450 nm was measured using an ELISA reader. The percentage of signal inhibition in relation to the negative control was calculated as Inhibition [%] = (1 − (Sample OD450/Average Negative Control OD450)) × 100. The inhibition cut-off, which was the positive cut-off provided in the SVNT kit, was 30%. An inhibition rate of ≥30% was considered positive for SARS-CoV-2 neutralizing antibodies, while <30% was considered negative.

### 2.5. Statistical Analysis

The collected data were analyzed using IBM-SPSS-Statistics V25.0 for Windows (IBM Corporation, New York, NY, USA). The anti-S-RBD results and the percentage of inhibition measured with CLIA and SVNT were reported in U/mL and %, respectively. The results were presented in the form of frequency tabulation. Furthermore, the correlation between CLIA and the % inhibition result of SVNT was determined using Spearman’s ranked test after the log transformation of the values. Based on the value of both tests, the receiver operating characteristic (ROC) for the detection of the specific levels of inhibition was determined. The sensitivity and specificity of CLIA were determined at a % inhibition cut-off of 30%. The cut-off protection point of CLIA was determined using the ROC curve, and the area under the curve (AUC) was measured.

## 3. Results

The result showed that 79 samples met the inclusion and exclusion criteria. The characteristics of the study subjects are shown in the Table 1.

The proportions of gender participation in this study were almost equal between female and male (57% vs. 43%), with the median age being 21–76 years old. The most prominent comorbidities were hypertension (15.2%) and type 2 diabetes mellitus (6.3%). The majority of subjects, 58 (73.4%), received two doses of COVID-19 vaccination, and 74 (93.7%) did not smoke. The majority, accounting for 34 (43%), were overweight with a BMI between 25 and 29.9. Meanwhile, 33 subjects (41.8%) were in the normal weight category with a BMI between 18.5 and 24.9. Table 1 shows a descriptive overview of the median as well as the minimum and maximum values of S-RBD SARS-CoV-2 IgG CLIA and SVNT collected from 79 samples. The result showed that the median of S-RBD SARS-CoV-2 IgG CLIA and SVNT was 3.18 and 46.91%, respectively. The time interval between vaccination and sample collection in this study ranged from 2 to 3 months

**Figure 1:**
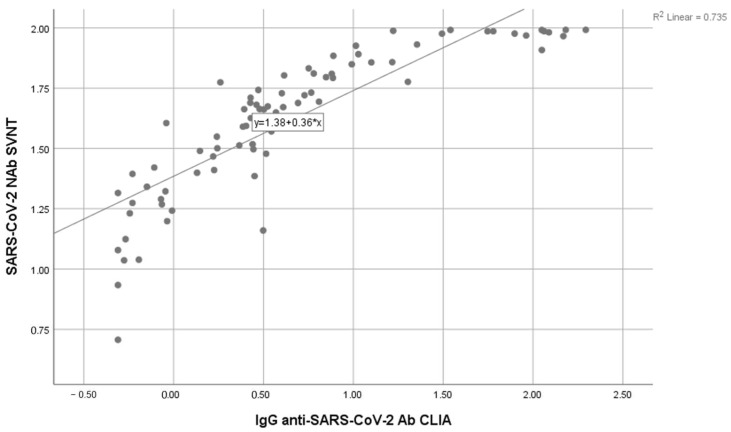
Correlation between the anti S-RBD antibody level using the CLIA and SVNT methods.

The correlation test between S-RBD SARS-CoV-2 IgG CLIA and SVNT was conducted using the Spearman ranked test (non-parametric; data not normally distributed) with a significance level of *p* < 0.05. The result showed *p* < 0.001, suggesting a significant correlation between S-RBD SARS-CoV-2 IgG CLIA and SVNT, with a positive direction. The correlation coefficient (r) of 0.933 (95% CI) showed a strong relationship. Therefore, the correlation coefficient provided in this study could be considered to detect antibody responses in SARS-CoV-2. Figure 1 shows the data distribution of SARS-CoV-2 NAb SVNT and IgG anti-SARS-CoV-2 Ab CLIA with log transformation.

In constructing the ROC curve, SVNT data were categorized as protective and non-protective when the value was ≥30% and <30%, respectively. S-RBD SARS-CoV-2 IgG CLIA values remain based on numerical data. Table 2 shows that a value ≥1.71 U/mL is the cut-off, followed by sensitivity and specificity of 96.5% and 90.9%, respectively, in diagnosing or predicting the protectiveness of S-RBD SARS-CoV-2 IgG CLIA, given that the protective threshold of SVNT is ≥30% inhibition. The measurement of a specific antibody, S-RBD SARS-CoV-2 IgG CLIA, is in U/mL.

According to the manufacturer, 1 U/mL is equivalent to 21.8 binding antibody units (BAU)/mL. Therefore, the cut-off obtained in this process was 37.29 BAU/mL. This is further supported by cross-tabulation results showing that there are 55 samples with the category of S-RBD SARS-CoV-2 IgG CLIA ≥ 37.29 BAU/mL and protective SVNT (≥30%). Additionally, 2 samples were found in the category of S-RBD SARS-CoV-2 IgG CLIA ≥ 37.29 BAU/mL and non-protective SVNT (<30%). Table 2 shows that the positive and negative values are 96.5% and 90.9% respectively.

**Figure 2:**
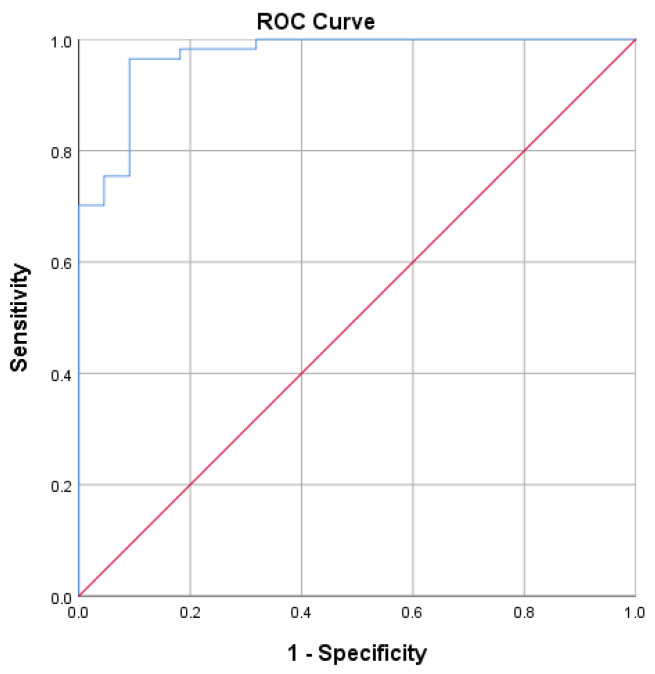
Receiver operating characteristic curve for S-RBD SARS-CoV-2 IgG CLIA. Area under the curve: 0.970. An ROC curve lying on the diagonal line reflects the performance of a diagnostic test (Blue Line) and the chance level (Red Line).

The AUC value of 0.970 in Figure 2 shows that S-RBD SARS-CoV-2 IgG CLIA data have no discrimination when connected with SVNT and are outstanding in suggesting protectiveness.

## 4. Discussion

The result of this study showed that the majority of subjects who met the inclusion criteria were female, with an average age of 48 years. A study conducted by Qi et al. reported that females had a faster and stronger anti-inflammatory response compared to males [13]. Zeng et al. also reported that IgG antibodies were produced more robustly in female subjects [14]. Bayram et al. showed that seropositivity was higher among females (84.6%) than males (70.6%) after the first dose (1D) of CoronaVac [15]. Moreover, Li et al. evaluated the effectiveness and immunogenicity of the three inactivated COVID-19 vaccines, in which female participants had significantly higher concentrations of SARS-CoV-2-specific spike (S) IgG and neutralizing antibodies than male participants [16]. Studies have shown that several immune cells, such as B lymphocytes, contain estrogen receptors regulated by estrogen levels. In fact, estrogen has been shown to promote immunoglobin production, while testosterone can inhibit it [17]. Antibody levels were found to be higher in younger adults compared to older adults, consistent with Khoury et al. [1]. In general, the number and function of naïve B and T cells in older individuals are reduced, resulting in weakened immunity to neo-antigens. Studies have shown that aging immune cells can generate a sufficient primary antibody response, although at a slower rate and with a lower ability to neutralize pathogens [18].

Recent evidence indicates that smokers exhibit lower antibody levels in response to COVID-19 mRNA vaccines, regardless of smoking duration or daily cigarette consumption. However, the underlying pathophysiological mechanisms explaining how smoking affects the dynamics of vaccine-induced anti-SARS-CoV-2 antibodies remain unclear. Smoking exposure compromises the immune system’s ability to generate memory cells crucial for sustaining protective immune responses triggered by vaccines. It is noteworthy that human IgG subclasses and specific antibodies typically have a half-life of around 3–4 weeks, depending on their attributes and IgG isotype. Moreover, cigarette smoking is linked to increased counts of monocyte-macrophage cells, which could potentially impact the clearance of antibodies circulating in the body [19].

Lower levels of SARS-CoV-2 antibodies were observed in subjects with comorbidities, such as hypertension, diabetes mellitus, and other chronic diseases. This result was consistent with Soegiarto et al. and Soetedjo et al. (2022), stating that subjects with comorbidities had lower levels of SARS-CoV-2 antibodies [20,21]. Recent studies indicate that hypertension correlates with systemic inflammation. Chronic systemic inflammation can lead to significant changes across tissues and organs, potentially affecting cellular functions, including immune responses to vaccines. Studies analyzing cellular subsets and profiles of inflammatory cytokines in this context have confirmed that heightened frequencies of activated innate immune cells and elevated levels of pro-inflammatory cytokines are associated with reduced responsiveness to vaccines [20].

The adaptive immune system may be compromised in diabetic patients due to impaired proliferation in response to antigenic stimulation, diminished production of CD4+ T follicular helper cells, and reduced capacity to generate effector lymphokines. Diabetic individuals often exhibit decreased numbers of circulating CD4+ cells, lower CD4+ to CD8+ lymphocyte ratios, impaired lymphocyte proliferative responses, and deficiencies in monocytes or macrophages, which impair antigen presentation. Interestingly, some studies have reported that patients with type 2 diabetes mellitus (T2DM) show elevated white blood cell counts, yet they are more likely to have decreased lymphocyte counts and an increased presence of senescent CD4+ and CD8+ T cells. These cells are characterized by heightened expression of chemokines, notably C-X-C motif chemokine receptor type 2, and exhibit altered migratory capabilities, contributing to poorer vaccine responses in diabetic patients. Furthermore, the hyperglycemic environment at the time of vaccination exacerbates the immunological response and further diminishes the immune system’s reaction to vaccines [22].

Severe obesity hastens the decrease in neutralizing antibodies following COVID-19 vaccination. Obesity has been linked to various abnormalities in B cells, such as an increase in B cells that produce pro-inflammatory cytokines. These existing dysfunctions in B cells are likely to contribute to a pro-inflammatory environment that could hinder the development of effective and long-lasting adaptive immune responses. Another crucial consideration is the potential changes in adipose tissue B cells in obese individuals, which might affect their ability to mount appropriate immune responses to SARS-CoV-2 infection or vaccination efforts. These findings have implications for vaccination strategies against SARS-CoV-2 variants and other infectious diseases among obese individuals [23]. Ali et al. discovered that fully vaccinated individuals who had previously contracted COVID-19 (natural immunity) exhibited significantly elevated levels of IgG and neutralizing antibodies compared to fully vaccinated individuals without prior infection (acquired immunity). Moreover, the study observed a more rapid decline in antibodies over time among those without previous infection, a finding consistent with earlier study [24].

This study aimed to evaluate the protective antibody levels against SARS-CoV-2 S-RBD using the CLIA compared to the SVNT method. It provides a reference that can be used for public health decisions, assessing the need for supplementary vaccination, and determining the time interval between vaccinations. In our country, the majority of the population was vaccinated with the Sinovac inactivated virus vaccines. Some studies showed that the subjects who received inactivated virus vaccines had lower antibody titer compared to subjects who received mRNA vaccines [25]. The lower antibody titers elicited by inactivated virus vaccines would be of even greater clinical concern for certain vulnerable groups of patients and health workers [26].

SVNT was shown to be a good surrogate test for PRNT and applicable in detecting the presence of neutralization antibody against SARS-CoV-2. The presence of neutralizing antibodies showed that the immune system recognized the virus and developed a neutralizing response essential for preventing infection and reducing the disease severity [27]. This study used the Genscript SVNT as the gold standard for evaluation due to the lack of availability of PRNT. A strong correlation was found between S-RBD SARS-CoV-2 IgG CLIA and SVNT methods. This result is consistent with the studies of Tiwari et al. in India, as well as Takahashi et al. and Kitagawa et al. in Japan, showing a strong correlation between CLIA and SVNT [28,29,30]. Furthermore, a value ≥37.29 BAU/mL was the cut-off, accompanied by a sensitivity of 96.5% and specificity of 90.9%, in predicting the protectiveness of IgG CLIA.

In this study, there were two patients with a S-RBD SARS-CoV-2 IgG CLIA level greater than or equal 37.29 BAU/mL, but the SVNT test results inhibition rate was less than 30%, which was considered negative for SARS-CoV-2 neutralizing antibodies. For the opposite, there were two patients with a S-RBD SARS-CoV-2 IgG CLIA level less than 37.29 BAU/mL, but the SVNT test results inhibition rate was more than 30%, which was considered positive for SARS-CoV-2 neutralizing antibodies. This could be due to several interference factors. The most common endogenous interferences include rheumatoid factors, heterophile antibodies and complement and cross-antigens. Exogenous interference mainly arises from incomplete coagulation or sample contamination [31]. Patients with connective tissue diseases can have high levels of ACE-2 antibodies, leading to false-positive SVNT results. False-positive results in EIA and SVNT tests have been linked to prior infections with seasonal coronaviruses [32]. A study on samples collected before the COVID-19 pandemic reported cross-reactions to acute infections with various pathogens, such as *Rickettsia typhi*, *Salmonella typhi*, *Leptospira* spp., and influenza B virus [33]. The connection between RBD and ACE-2, where neutralizing antibodies bind most frequently, is affected by antigenicity modification that can also contribute to this phenomenon [9]. Due to the high SARS-CoV-2 mutation rate, the circulating strains are constantly evolving and changing in terms of their antigenicity [34]. Another cause of this phenomenon is because the SVNT method cannot detect all neutralizing antibodies, only antibodies to RBD [35].

The measurement of S-RBD SARS-CoV-2 IgG CLIA can detect whether the measured anti-S-RBD means sufficient protection against COVID-19. Individuals with adequate protection could be offered additional vaccinations, specifically in an immunocompromised state. However, this study had limitations, including factors causing cross-reactivity in CLIA methods, such as human metapneumovirus (HMPV), common cold coronavirus, influenza virus, rhinovirus, metapneumovirus, and adenovirus, which were not identified [36]. Low antibody titers occurred in certain conditions, including sampling during the pre-seroconversion period, and a decrease in antibody titers over time, in elderly, immunosuppressed, and immunocompromised subjects [37]. The study from Zhang et al. showed that the neutralizing antibodies positivity rate was the highest at the first and second month after the second dose of vaccine and gradually decreased over time [38]. In this study, the time interval between vaccination and sample collection was not considered.

We acknowledge that the reference standard used in this study, the SVNT, is not the ideal gold standard. The most accurate method for determining neutralizing antibodies is the plaque reduction neutralization test (PRNT). However, performing the PRNT requires significant resources, including a laboratory with a high biosafety level. Another option is the pseudovirus neutralization test (PVNT), which shows a higher correlation with tests based on live viruses. Nevertheless, several studies have demonstrated a strong correlation between PVNT and SVNT results, as well as the reliability of SVNT in detecting neutralizing antibodies while being much more accessible to most laboratories [39].

A comparison between S-RBD SARS-CoV-2 IgG and PRNT should have been conducted, but this test is currently unavailable in Indonesia. By comparing S-RBD SARS-CoV-2 IgG CLIA to GenScript SVNT, an approximation can be provided regarding the predictive capability of CLIA in determining the level of protection. Hence, it is likely that vaccines will be administered seasonally, necessitating appropriate protocols. In scenarios where vaccine availability is restricted, prompt vaccination may be crucial for immunocompromised individuals and those who have not previously been infected, as they might remain vulnerable to infection post-vaccination and face a heightened risk of severe illness. Overall, guidance on the necessity for booster doses should involve ongoing monitoring of antibody levels in vaccinated individuals to ensure a sustained protective immune response against COVID-19.

## 5. Conclusions

In conclusion, this study found a strong correlation between S-RBD SARS-CoV-2 IgG CLIA and SVNT. The AUC value showed 0.970, suggesting that CLIA had excellent discrimination and reliability when connected with SVNT. Additionally, a titer of antibodies greater than or equal to 37.29 BAU/mL with the CLIA showed the presence of protective antibodies compared to SVNT.

## Figures and Tables

**Table 1:** Characteristics of the subjects.

Characteristics		Frequency (*n* = 79)	Percentage (%)
Age, (years)			
Median	48		
Min–Max	21–76		
Sex			
Male		34	43
Female		45	57
History of Smoking			
No		74	93.7
Yes		5	6.3
Comorbidity			
History of Chronic Illness		19	24.1
Hypertension		12	15.2
Type 2 Diabetes Mellitus		5	6.3
Asthma Bronchiale		3	3.8
Chronic Obstructive Pulmonary Disease		1	1.3
Body Mass Index (kg/m^2^)			
Underweight (<18.5)		1	1.3
Normal weight (18.5–24.9)		33	41.8
Overweight (25–29.9)		34	43
Obese (≥30)		11	10.1
Complete COVID-19 Vaccination *		58	73.4
Previous COVID-19 Infection		2	2.5
IgG anti-SARS-CoV-2 Ab CLIA (U/L)			
Median	3.18		
Min–Max	0.49–196.66		
SARS-CoV-2 SVNT (%)			
Median	46.91		
Min–Max	5.09–98.19		

* Complete vaccination equals two times vaccination (based on Indonesian Health Protocol in 2020).

**Table 2:** The diagnostic accuracy of S-RBD SARS-CoV-2 IgG CLIA cut-off point for the 30% inhibition level of SVNT.

	SVNT					
S-RBD SARS-CoV-2 IgG CLIA (BAU/mL)	Protective (≥30%)	Non-Protective (<30%)	Sensitivity (%)	Specificity (%)	Positive Predictive Value (%)	Negative Predictive Value (%)
≥37.29	55	2		90.5		
<37.29	2	20	96.5		96.5	90.9
Total	57	22				

## Data Availability

The data used to support the result of this study were included in the article.
